# Transcriptional regulation of *KCS* gene by bZIP29 and MYB70 transcription factors during ABA-stimulated wound suberization of kiwifruit (*Actinidia deliciosa*)

**DOI:** 10.1186/s12870-021-03407-6

**Published:** 2022-01-08

**Authors:** Xueyuan Han, Xiaopeng Wei, Wenjing Lu, Qiong Wu, Linchun Mao, Zisheng Luo

**Affiliations:** 1 School of Life Science, Shaoxing University, Shaoxing, 312000 Zhejiang Province China; 2 College of Biosystems Engineering and Food Science, Zhejiang Key Laboratory of Agro-Food Processing, Key Laboratory of Agro-Products Postharvest Handling of Ministry of Agriculture and Rural Affairs, Zhejiang University, Hangzhou, 310058 China; 3 College of Food and Bioengineering, Zhengzhou University of Light Industry, Zhengzhou, 450002 Henan China; 4 Institute of Food Science, Zhejiang Academy of Agricultural Sciences, Hangzhou, 310021 China; 5 College of Food Science and Engineering, Henan University of Technology, Zhengzhou, 450001 China; 6 Ningbo Research Institute, Zhejiang University, Ningbo, 315100 China

**Keywords:** Suberization, KCS, Transcriptional regulation, Abscisic acid

## Abstract

**Background:**

Our previous study has demonstrated that the transcription of *AchnKCS* involved in suberin biosynthesis was up-regulated by exogenous abscisic acid (ABA) during the wound suberization of kiwifruit, but the regulatory mechanism has not been fully elucidated.

**Results:**

Through subcellular localization analysis in this work, AchnbZIP29 and AchnMYB70 transcription factors were observed to be localized in the nucleus. Yeast one-hybrid and dual-luciferase assay proved the transcriptional activation of AchnMYB70 and transcriptional suppression of AchnbZIP29 on *AchnKCS* promoter. Furthermore, the transcription level of *AchnMYB70* was enhanced by ABA during wound suberization of kiwifruit, but *AchnbZIP29* transcription was reduced by ABA.

**Conclusions:**

Therefore, it was believed that ABA enhanced the transcriptional activation of AchnMYB70 on *AchnKCS* by increasing *AchnMYB70* expression. On the contrary, ABA relieved the inhibitory effect of AchnbZIP29 on transcription of *AchnKCS* by inhibiting *AchnbZIP29* expression. These results gave further insight into the molecular regulatory network of ABA in wound suberization of kiwifruit.

**Supplementary Information:**

The online version contains supplementary material available at 10.1186/s12870-021-03407-6.

## Background

Fruits are often bruised or mechanically wounded during the harvesting, transportation and storage processes, which leads to the susceptibility to microbial infection and quality degradation. However, the damaged surface of the postharvest kiwifruit would suberize to accumulate suberin and further form a healing layer, which can reduce the outflow of cell water and nutrients and limit the invasion of pathogens [1–3]. Suberin layer was observed after wounding by means of fluorescence and staining microscopy and component analysis in kiwifruit [1]. Wounding-induced suberization also commonly occurs in potato tuber [4], *Arabidopsis* root [5] and postharvest tomato [6]. Suberin is a plant cell-wall biopolymer composed of glycerol-based aliphatic polyester and the associated polymeric aromatics [7, 8]. It is biosynthesized initially from the acylation of fatty acids by long chain acyl-CoA synthetase (LACS), following fatty acyl elongation controlled by fatty acid elongation enzyme complex (FAE), acyl reduction by fatty acyl reductase (FAR), fatty acyl oxidation by cytochrome P450 enzyme (CYP) and esterification of ω-hydroxy fatty acids and α, ω-dicarboxylic acids by glycerol 3-phosphate acyltransferase (GPAT) [9]. The polymeric aromatics are biosynthesized from phenylpropanoid pathway [7].

**Fig. 1:**
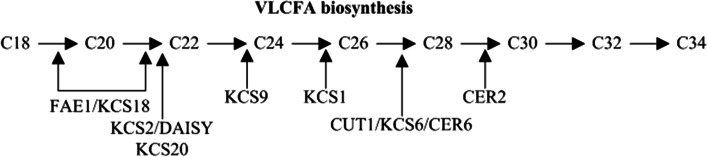
Catalysis and substrate specificity of KCSs in the elongation steps of carbon chains involved in the synthesis of VLCFAs in Arabidopsis [13]. Numbers represent the number of carbon units of VLCFAs

Exogenous abscisic acid (ABA) could stimulate the accumulation of suberin with induced expression of genes encoding β-ketoacyl-coenzyme A synthases (KCSs) related to suberin synthesis [1, 10]. It was suggested that ABA signaling stimulated the formation of a periderm including suberin in the apple and tomato fruit with defective cuticle formation [11, 12]. KCSs, as the components of FAE, catalyze the condensation of long-chain fatty acyl CoA and malonyl CoA to produce β-ketoacyl CoA with a carbon chain extension of two-carbon unit (Fig. 1), participating in the synthesis of very long chain fatty acids (VLCFAs) that are the precursors of suberin biosynthesis. Resent research also reported that KCSs were associated with peridermal skin formation in kiwifruit [14]. The coding sequence (CDS) of *AchnKCS (Achn030011)* of 1512 bp was cloned from *Actinidia deliciosa* ‘Xuxiang’ in our previous work [15]. The homology analysis of amino acid sequence displayed that the KCSs in plant were highly conserved, and AchnKCS had a high homology with AtKCS20 in *Arabidopsis* [16] and SlKCS11 in tomato [17]. In addition, the endoplasmic reticulum (ER) localization of AchnKCS protein was confirmed [15].

QsMYB1 (*Quercus suber*) was reported to target two *QsKCS* involved in suberin biosynthesis by Chip-seq assay [18]. Recently, it was revealed that AchnbZIP12 responding to ABA signaling positively regulated the transcription of *AchnKCS* during wound suberization of kiwifruit [15]. AtMYB41 [19], AtMYB9 [12], AtMYB107 [20] and AtMYB93 [21, 22] were demonstrated to be associated with the regulation of suberin biosynthesis. The over expression of *MYB92* in leaves of *Nicotiana benthamiana* significantly increased the transcript level of *KCS1* and the deposition of corresponding suberin monomers with carbon chain length of > 20 [23]. Similarly, the transcript levels of *KCS2* and *KCS20* were elevated in *MYB39* overexpression leaves of *N. benthamiana*, and *KCS1* and *KCS2* in *MYB39* overexpression root of Arabidopsis [24]. Moreover, some of these transcription factors involved in suberization regulation have been shown to be ABA-responsive, such as AtMYB41 [19], AchnbZIP12 [15] and AchnMYB107 [25]. Besides, ABA signaling cascades was suggested to play a mediating role in suberin biosynthesis regulated by MYB39 in the Arabidopsis root endodermis [24].

Therefore, based on our previous report and related literatures, the present study was to explore the regulatory mechanism of ABA in inducing *AchnKCS* (*Achn030011*) expression during suberin deposition by investigating the transcriptional control of transcription factors on *AchnKCS*. AchnbZIP29 and AchnMYB70 transcription factors were speculated and verified to regulate the transcription of *AchnKCS* in respond to ABA-stimulated wound suberization. It was expected to give further insight into the molecular regulatory network of ABA in promoting wound suberization of kiwifruit.

## Methods

### Fruit treatment

Kiwifruit (*Actinidia deliciosa* ‘Xuxiang’) were harvested at commercial maturity with the uniformity of shape and size from a commercial orchard in Fuyang District, Hangzhou, China. Treatment was based on Han et al. [15]. The surface was sterilized with 0.5% (v/v) NaClO solution for 3 min, washed with sterile water and air-dried naturally. Artificial wound was made by cutting the fruit into halves lengthwise. Nighty halves were treated with 0.5 mmol L^− 1^ ABA (≥ 90%, Aladdin Industrial Inc., China) and another 90 halves were treated with sterile water (control) by vacuum infiltration. Afterwards, fruit halves were stored in a sterile incubator at 20 °C and 85% relative humidity for wound healing under darkness. Suberized tissue was separated from the scarred outmost layer of the wound surface after incubating for 2, 3 and 4 days and stored at − 80 °C until further analysis.

### RNA extraction

The cetyltrimethylammonium bromide (CTAB) method was carried out to extract the total RNA [26]. The implementation details referred to Han et al. [15]. Briefly, 2% CTAB extraction buffer and LiCl solution (12 mol L^− 1^) were applied to extract and denature the RNA on the first day. On the second day, the SSTE buffer (containing 1.0 mM EDTA, 10 mM Tris-HCl pH 8.0, 0.5% (m/v) SDS and 1.0 M NaCl), chloroform and ethanol were added to dissolve, purify and precipitate the RNA, respectively. Finally, wash the RNA pellet with pre-chilled 75% ethanol for twice and dissolve the RNA pellets again using RNase-free water. The quality of the RNA samples was measured using a NanoDrop 2000 (Thermo Fisher Scientific, USA).

### DNA extraction

The total DNA was extracted by implementing the CTAB method [27]. The implementation details referred to Han et al. [15]. Briefly, 2% CTAB buffer, the solution of phenol: chloroform: isoamylol (25:24:1) and the solution of chloroform: isoamylol (24:1) were applied to extract and purify the DNA. After centrifuging, NaAc solution and isopropanol were added to precipitate the DNA. Afterwards, wash and dissolve the DNA precipitate respectively with 75% (v/v) ethanol and TE buffer. The quality of DNA samples was measured by a NanoDrop 2000.

### Molecular cloning and amino acid sequence homology

The gene sequence of transcription factor AchnbZIP29 (Achn340751) and AchnMYB70 (Achn117821) were determined based on the Cornell University kiwifruit database (http://bioinfo.bti.cornell.edu/cgi-bin/kiwi/home.cgi). The cloning conditions were according to Han et al. [15]. Based on the primers in Supplementary Table 1 (*AchnbZIP29-Full* and *AchnMYB70-Full*), both genes of *AchnbZIP29* and *AchnMYB70* were cloned from reverse transcribed cDNA. And the promoter of *AchnKCS* was cloned from the extracted total DNA using the corresponding *AchnKCS-Pro* primers. After linking the amplified product with pEASY-T1 simple vector and transferring it into *Escherichia coli*, the test of white spot screening was carried out to obtain the recombinant plasmid.

The cloned sequence was compared with the proteins of *Arabidopsis thaliana* on NCBI BLAST software, Then the sequences with the high identified score were downloaded and multiple sequence alignment were further carried out by means of DNAMAN8 (Lynnon Biosoft Corporation, USA). The corresponding phylogenetic tree was mapped using MEGA7 software (www.megasoftware.net/).

### Subcellular localization of AchnbZIP29 and AchnMYB70

After cloning the coding sequence (CDS) of *AchnbZIP29* and *AchnMYB70*, the sequence with no stop codon was amplified and inserted into the 1300-35S-eGFP vector. The obtained AchnbZIP29-GFP and AchnMYB70-GFP fusion expression vectors were respectively transferred into *Agrobacterium* strain. The preparation of the infection buffer of *Agrobacteria* and the inoculation of tobacco (*Nicotiana benthamiana*) leaves were according to Han et al. [15]. After inoculation for 48 h, a confocal microscope (Leica SP8, Leica Microsystems Co., Germany) was used to observe the GFP fluorescence of the leaf discs at 488 nm excitation.

### Yeast one-hybrid assay (Y1H)

In order to test the protein-DNA interaction of AchnbZIP29, AchnMYB70 and *AchnKCS* promoter, Y1H assay was carried out according to the Matchmaker® Gold Yeast One-Hybrid Library Screening System (Cat. No. 630491, TaKaRa, Dalian, China). Auto-activation analysis of *AchnKCS* promoter was conducted at first and the minimum inhibitory concentration of aureobasidin A (AbA, a yeast toxin) was determined. The recombinant plasmid of AchnKCS-Pro-pABAi was transferred into Y1H Gold through PEG/LiAc after linearizing. The full-length regions of *AchnbZIP29* and *AchnMYB70* were cloned into pGADT7 vector (AD) via restriction enzyme cutting sites (*EcoR*I and *Xho*I sites, *Sma*I and *Sac*I sites, respectively). Transformed Y1H Gold harboring both AchnKCS-Pro-pABAi and AchnbZIP29-pGADT7 or AchnMYB70-pGADT7 were cultured to test the interaction on SD/−Leu with AbA at 30 °C for 3 days. Y1H Gold co-transformed with p53-promoter and pGADT7-Rec were used as positive control. Y1H Gold co-transformed with AchnKCS-Pro-pABAi and empty pGADT7 were used as negative control.

### Dual luciferase assay

Dual-luciferase assay was carried out to determine the trans-activation role of AchnbZIP29 and AchnMYB70 on target *AchnKCS* promoter. The implementation details referred to Tao, et al. [28]. The promoter sequence of *AchnKCS* was inserted into LUC vector (pGreen II 0800-LUC, cut by *Hind*III and *BamH*I). The CDSs of *AchnbZIP29* and *AchnMYB70* were amplified and inserted into pGreen II 0029 62-SK vector (SK) (cut by *Hind*III and *BamH*I), respectively. The ClonExpress II One Step Cloning Kit (C112–01, Vazyme, China) was applied to drive the connection reactions. The procedures of *Agrobacterium tumefaciens* transformation and the preparation of the infection buffer of *Agrobacteria* were according to Han et al. [15]. Afterwards, the *Agrobacteria* culture mixtures of respectively empty pSK, AchnbZIP29-pSK or AchnMYB70-pSK and *AchnKCS* promoter-pLUC (v/v 10:1) were prepared to infect tobacco (*Nicotiana benthamiana*) leaves with needleless syringes. A total of three tobacco plants were used and two leaves of each plant were selected for infection. That was six biological replicates were considered to determine the results. After 72 h for infiltration, the Dual-Luciferase Reporter Assay System (E1910, Promega, USA) with Modulus Luminometers (Promega, USA) was employed to detect the activities of firefly luciferase (LUC) and renilla luciferase (REN).

### Real-time quantitative reverse transcription PCR analysis (qRT-PCR)

The first-strand cDNA was obtained by RNA reverse transcription according to the manufacturer’s instructions of PrimeScript™ RT reagent Kit (Perfect Real Time, TaKaRa Bio Inc., China). The CFX96-TouchTM Deep Well Sequence Detection system (Bio-Rad Laboratories, Inc. CA, USA) was applied to detect gene transcription levels with SYBR® *Premix Ex Taq™*II (TliRNaseH Plus, TaKaRa Bio Inc., China). Each gene was analyzed in triplicate and *Actin* was used as reference gene. The relative expression levels of genes were calculated by the 2^-△△CT^ method [29] and presented in multiples relative to the initial value without any treatment (normalized to 1).

### Statistical analysis

Each experiment included at least three biological replicates. Data represented the mean value minus or plus standard deviation (± SD). SPSS software (version 20.0, IBM Corporation, New York, America) was used to analyze the difference significance by Least significant difference (LSD) test and Origin 9.0 software (OriginLab Corporation, Massachusetts, America) for mapping. The difference was considered to be statistical significance when *p* ≤ 0.05 or 0.01, and expressed with different letters or “*”, “**” in figures.

## Results

### Analysis of *AchnKCS* promoter sequence

Based on the total DNA template of kiwifruit, a 709 bp sequence of *AchnKCS* promoter was successfully amplified by the primer of *AchnKCS-Pro-F/R* in Supplementary Table 1. The sequence analysis through PlantCARE software (http://bioinformatics.psb.ugent.be/webtools/plantcare/html) showed that *cis*-acting elements of ABRE (ABA responsive element), G-box, MBS and MRE were contained (Table 1). ABRE was considered to be specifically recognized by bZIP transcription factors and involved in ABA response, while G-box was supposed as coupling of ABRE [30, 31]. MBS and MRE were the binding sites of MYB transcription factors [32].

**Table 1:** Bioinformatic analysis of *AchnKCS* promoter

Element	Description	Sequence (5′-3′)	Position
ABRE	*cis*-acting element involved in the abscisic acid responsiveness	TACGTG	− 1025(+)
ABRE	*cis*-acting element involved in the abscisic acid responsiveness	TACGTG	− 1498(−)
G-Box	*cis*-acting regulatory element involved in light responsiveness	CACGTT	− 1587(−)
G-Box	*cis*-acting regulatory element involved in light responsiveness	CACGTC	− 961(−)
MBS	MYB binding site involved in drought-inducibility	CAACTG	− 1203(−)
MRE	MYB binding site involved in light responsiveness	AACCTAA	− 999 (+)

Note: Position represents the *cis*-acting element is counted from the position of ATG

### Amino acid sequence homology

**Fig. 2:**
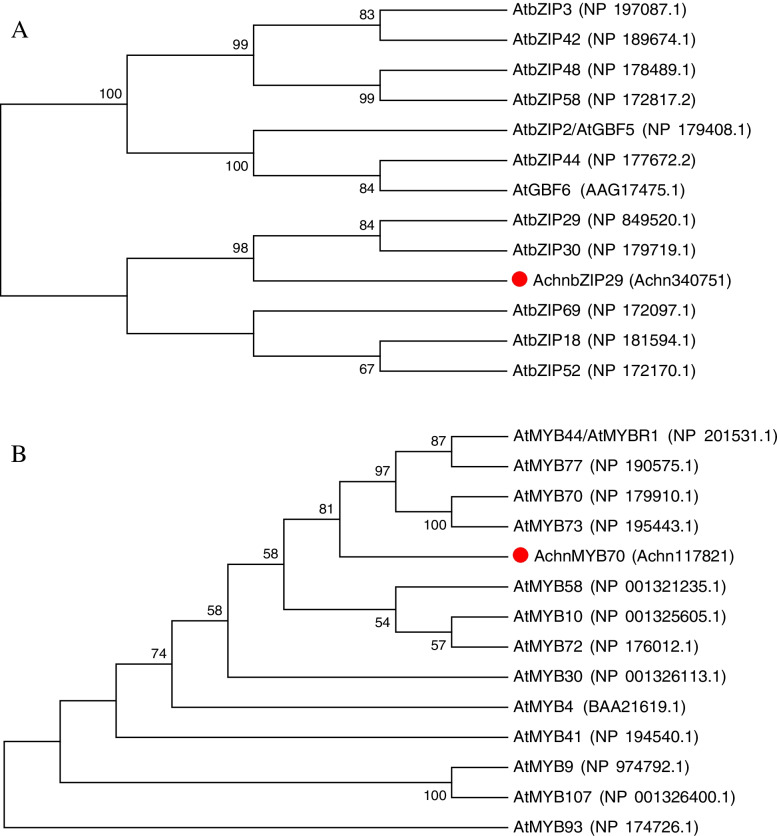
Amino acid sequence phylogenetic analysis of AchnbZIP29 and AchnMYB70 from kiwifruit and bZIP and MYB members from Arabidopsis. The amino acid sequences were obtained from the Cornell University kiwifruit database and NCBI database, respectively. The accession numbers were indicated in the brackets

Through the promoter sequence analysis by PlantCARE and bioinformatics searching by NCBI BLAST software, a bZIP (Achn340751) and an MYB (Achn117821) transcription factor were inferred to be downstream responses of ABA signaling and be associated with suberin biosynthesis based on the involvement of their close homologs in ABA responding and mechanical stress [33–38]. Using cDNA as template, the CDS of *Achn340751* and *Achn117821* were cloned. Furthermore, the BLAST online software was used to analyze the sequence homology from the NCBI database. Based on its homology with Arabidopsis transcription factors presented as phylogenetic tree by means of DNAMAN8 and MEGA7 software in Fig. 2, they were temporarily designated as AchnbZIP29 and AchnMYB70. And it showed that AchnbZIP29 and AchnMYB70 respectively belonged to Group I of bZIP transcription factors and R2R3-MYB 22 subgroup, which involved in the regulation of fatty acid biosynthesis [39–41].

### Subcellular localization

**Fig. 3:**
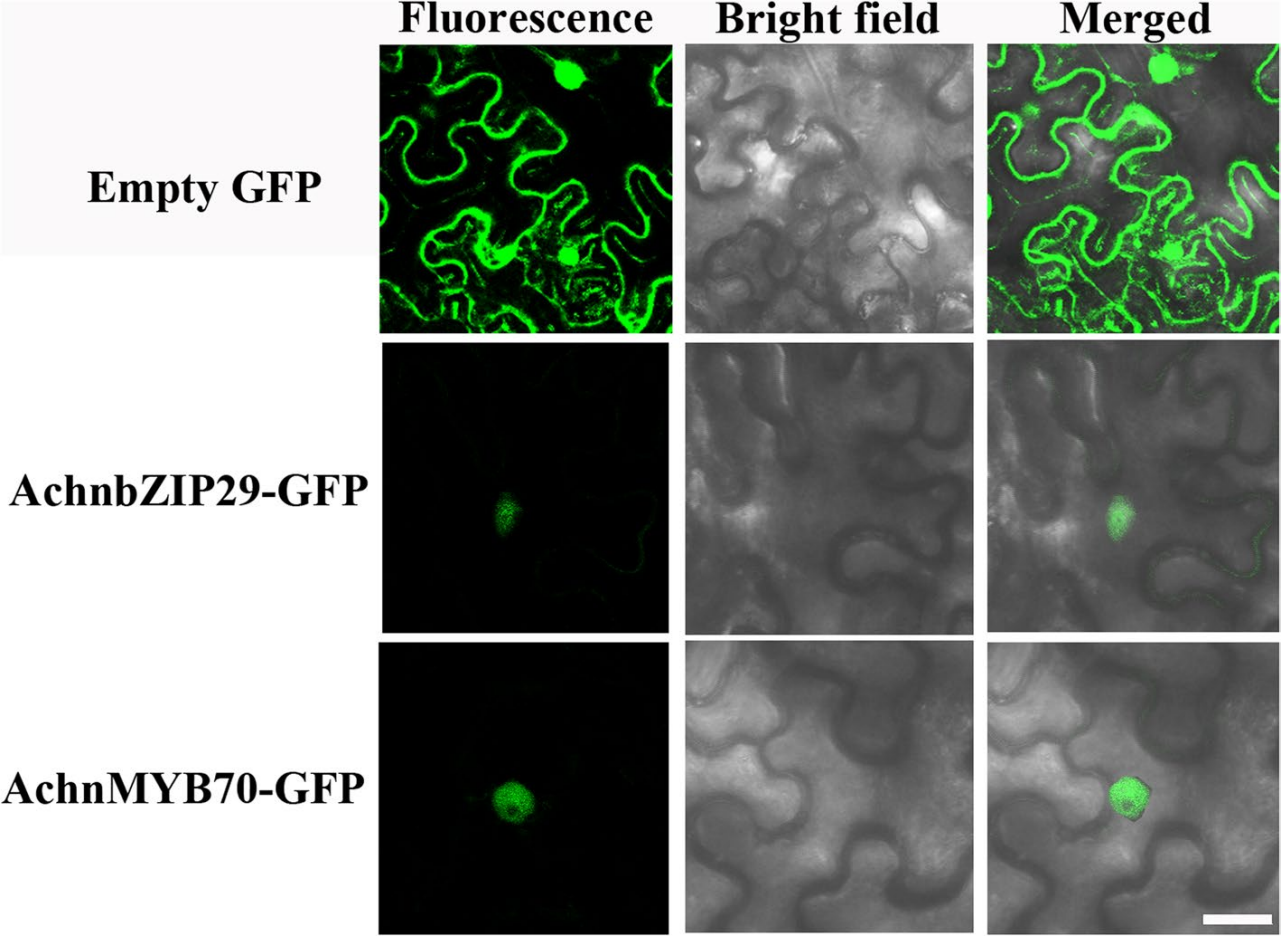
Subcellular localization of AchnbZIP29 and AchnMYB70 indicated by GFP green fluorescence in *Nicotiana benthamiana* epidermal cells. Bars = 50 μm

In order to speculate the functional mechanism, the subcellular localization of both transcription factors was determined by observing the fluorescence signal of GFP based on the fusion expression vectors of the reporter gene *GFP* with *AchnbZIP29* or *AchnMYB70*. The result displayed that compared with the green fluorescence appearing in the whole cell of the hollow GFP vector, the GFP green fluorescence signal of the fusion expression vector with the AchnbZIP29 or AchnMYB70 appeared specifically in the nucleus (Fig. 3). It indicated that AchnbZIP29 and AchnMYB70 were located in the nucleus, conforming their functional characteristics of regulating gene transcription.

### Interaction between AchnbZIP29, AchnMYB70 and *AchnKCS* promoter

**Fig. 4:**
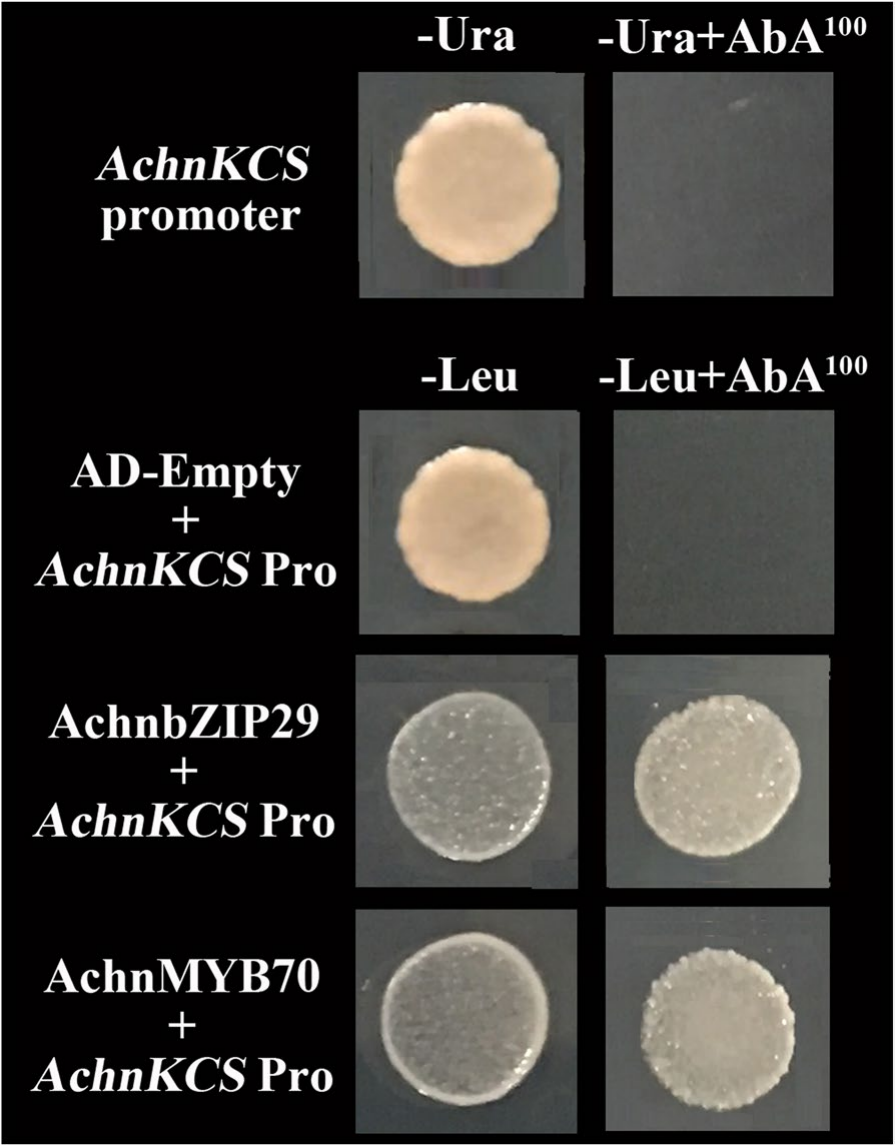
Yeast one-hybrid analysis on interaction between AchnbZIP29, AchnMYB70 and *AchnKCS* promoter

Y1H was carried out to investigate whether AchnbZIP29 and AchnMYB70 can interact with *AchnKCS* promoter. Firstly, the self-activation test showed that the yeast transformed with AchnKCS-Pro-pABAi cannot grow on the medium containing 100 ng mL^− 1^ AbA (Fig. 4). Subsequently, Y1H displayed that the positive control strain (AD-Rec-p53 + p53 promoter, not shown) and Y1HGold transformed with AchnbZIP29 + *AchnKCS* Pro, and AchnMYB70 + *AchnKCS* Pro can grow in the medium with 100 ng mL^− 1^ AbA and no leucine (−Leu) (Fig. 4), which verified the interaction of individually AchnbZIP29 and AchnMYB70 with *AchnKCS* promoter.

**Fig. 5:**
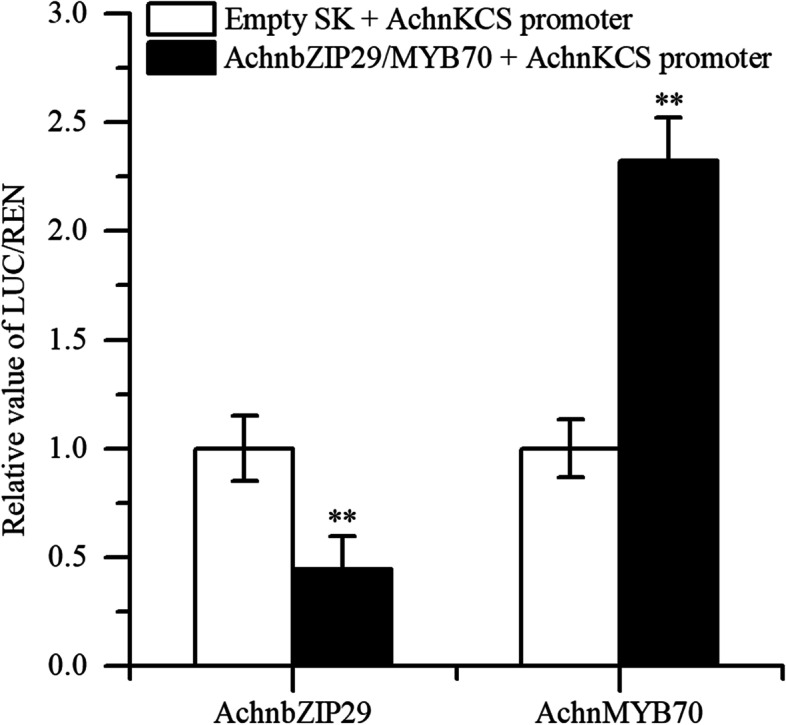
The transcriptional effect of AchnbZIP29 and AchnMYB70 on the promoter of *AchnKCS* by dual-luciferase assay. The LUC/REN value for the empty vector (SK) was set as 1

Besides, in order to further clarify the regulatory effect of AchnbZIP29 and AchnMYB70 on *AchnKCS*, a dual luciferase assay was applied. It presented that AchnMYB70 can significantly enhance the transcriptional activity of *AchnKCS* promoter, and the ratio of LUC/REN was 2.32 times that of the control (SK) (Fig. 5). In contrast, AchnbZIP29 negatively regulated the transcriptional activity of *AchnKCS* promoter, and its LUC/REN ratio was only 0.44 that of SK (Fig. 5).

### Effect of exogenous ABA on the transcription levels of *AchnbZIP29* and *AchnMYB70*

**Fig. 6:**
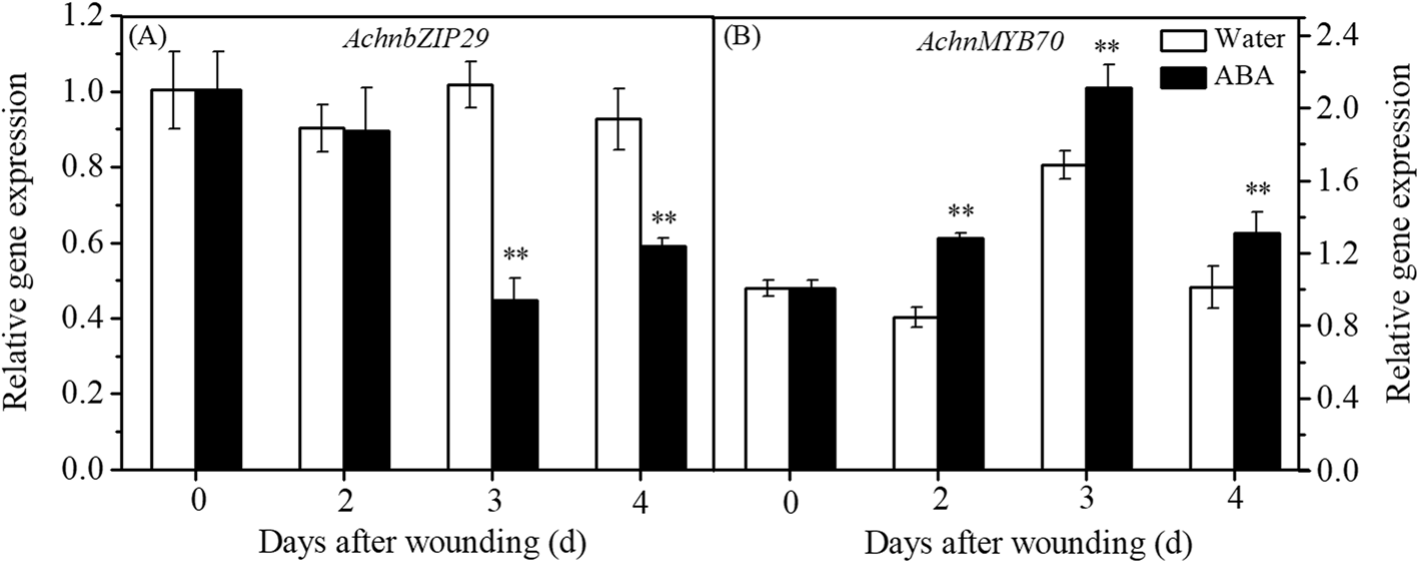
Relative transcription levels of *AchnbZIP29* and *AchnMYB70* during wound suberization of kiwifruit. “**” represents significant difference at *p* ≤ 0.01

The relative transcription levels of *AchnbZIP29* and *AchnMYB70* in ABA-stimulated suberizing tissue of kiwifruit were analyzed by qRT-PCR. As shown in Fig. 6, the transcription level of *AchnbZIP29* was reduced by exogenous ABA and decreased to 0.45 of the initial value (normalized to 1) on the third day after treatment. On the contrary, the transcription level of *AchnMYB70* was significantly up-regulated by ABA. From the second day after treatment, the transcription level of *AchnMYB70* in the suberizing tissue increased significantly and reached the maximum abundance on the third day, which was 2.1 times of the initial control value. The difference in relative transcript abundance induced by ABA further illustrated that AchnbZIP29 and AchnMYB70 were ABA signal-responsive transcription factors.

## Discussion

Abscisic acid (ABA) is a stress resistance hormone in plant, which is involved in a variety of biotic and abiotic stresses [42, 43]. Relevant studies in recent years have shown that ABA promoted suberin accumulation in Arabidopsis root [5, 43], potato tuber [4, 44], tomato fruit [6, 45] and kiwifruit [1]. Wounding also induced the increase of ABA level in potato tuber [4]. The increased expression of genes in suberin pathway with an ABA-dependent manner in russet apple further suggested the important role of ABA signaling in suberin development [11]. Moreover, the inhibition of ABA biosynthesis by fluridone was reported to block the wound suberization in potato tuber [4] and tomato fruit [6]. ABA has been verified to be a positive regulator in suberin deposition and confirmed the role in wound suberization of kiwifruit [1, 6, 46]. In detail, ABA treatment could induce suberin precursor VLCFAs accumulation during wound suberization [4, 47, 48]. In VLCFAs biosynthesis, KCSs are the rate-limiting enzymes in the chain elongation of fatty acids [49]. It was further found that the *KCS* gene was significantly induced in response to ABA-stimulated suberization of kiwifruit [15].

The promoter sequence of ABA-responsive genes generally has a conserved *cis*-acting element, namely ABA-responsive element (ABRE; PyACGTGG/TC) [50, 51]. Transcription factors of bZIP family in plant could interact with *cis*-acting elements containing ACGT sequence to participate in ABA signaling [52–54]. In Arabidopsis, it has identified eighty bZIP transcription factors, which are divided into 13 groups based on the similarity of their basic regions and other conserved motifs [55]. It was reported that AchnABF2 and AchnbZIP12 in Group A responding to ABA activated the transcription of *AchnFHT* and *AchnKCS* involved in suberin biosynthesis, respectively [15, 25]. In this work, *AchnbZIP29* was cloned from kiwifruit and the analysis of amino acid sequence showed that it was classified into Group I. The bZIPs of Group I in Arabidopsis were likely to be involved in the development of vascular tissue and cell wall [56]. AchnbZIP29 presented high homology with AtbZIP29. Related research revealed that ABA decreased the expression of AtbZIP29 in guard cells [57]. Similarly, the transcription level of *AchnbZIP29* was down-regulated by ABA during wound suberization in this work. It was also speculated that AtbZIP29 regulated the expression of CYP707A3 and CYP707A1 which were two key enzymes involved in ABA catabolism [37]. Accordingly, it was inferred that AchnbZIP29 negatively correlated with the expression of ABA-responsive genes and it was likely to participate in the regulation of wound suberization on the cell wall, but its target gene was possible not only *AchnKCS*.

However, *cis*-acting element alone was not sufficient for regulating the transcription of ABA-responsive genes. The interaction between AREB (ABRE binding proteins) and ABRE required the participation of coupling elements [58]. Considered as a coupling element of ABRE motif, the G-box element was reported to play roles in regulating gene expression under various environmental stresses [59]. Certain bZIP transcription factors contained motifs that recognized and bound to G-box element [30, 59]. In this work, the cloned *AchnKCS* promoter region contained not only two ABRE elements, but also two G-box elements. It allowed us to further determine that bZIP transcription factors played an important regulatory role in the ABA-promoted suberization.

MYB transcription factor family has a wide range of function diversity, including the regulation of suberin biosynthesis [12, 19, 21]. In this work, AchnMYB70 was found to activate the *AchnKCS* promoter and positively regulate the *AchnKCS* transcription. Most MYB proteins bound to one or more cis-acting elements (MBS/MRE) with the conserved sequence of CNGTT(A/G) or C(G/T)T(A/T) GTT(A/G) [32]. It showed that AchnMYB70 had high homology with AtMYB70, AtMYB73 and AtMYB44, which were involved in secondary metabolism and resisting biotic and abiotic stress in Arabidopsis [33, 60, 61]. The lipid content in seeds and leaves of transgenic Arabidopsis overexpressing the *GmMYB73* (*Glycine max*) gene was significantly increased [40]. It was also reported that osmotic stress induced the transcription of *AtMYB30* and *AtMYB4*, which was associated with the FAE complex and contributed to the synthesis of VLCFAs [62]. In addition, *AchnMYB107* and *AchnMYB41* were induced by exogenous ABA during wound suberization of kiwifruit and were demonstrated to activate the transcription of *AchnFHT*, *AchnFAR* and *AchnCYP86A1* that were involved in suberin biosynthesis [25, 46, 63]. In this study, the transcription level of *AchnMYB70* was also up-regulated by exogenous ABA treatment and was proved to possibly have an activating effect on *AchnKCS* transcription during wound suberization of kiwifruit.

The transcription of a gene may be comprehensively regulated by multiple transcription factors, and the interaction between transcription factors may jointly play a role in the transcriptional regulation of the target genes. In this work, any interaction or other cooperative regulation between the transcription factors that can interact with the *AchnKCS* promoter, including AchnbZIP29, AchnMYB70 and AchnbZIP12 we reported previously, still needed to be further studied.

## Conclusions

**Fig. 7:**
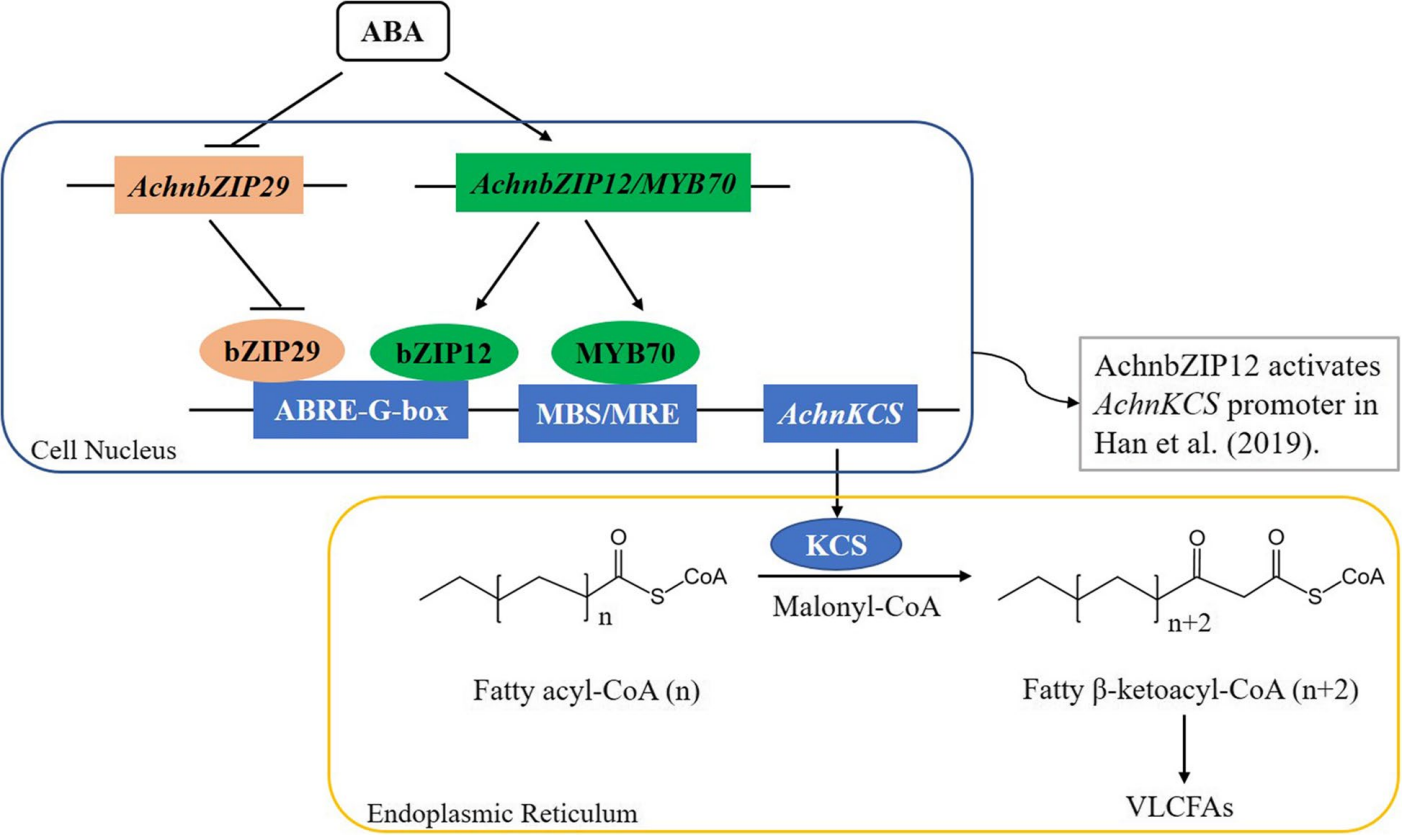
The model of the transcriptional regulation of AchnbZIP29, AchnMYB70 and AchnbZIP12 on *AchnKCS* responding to ABA during wound suberization of kiwifruit. Note: AchnbZIP12 and AchnMYB70 induced to increase *AchnKCS* transcription through interacting with *cis*-acting element (ABRE/G-box and MBS/MRE). The down-regulated transcription of *AchnbZIP29* relieved the inhibitory effect of AchnbZIP29 on *AchnKCS*. And KCS as the key component of FAE complex catalyzed the chain elongation of fatty acyl-CoA (Cn ≥ C16) to fatty β-ketoacyl-CoA (C(n + 2)), further producing VLCFAs that were precursors of suberin

In conclusion, the present work explored a potential regulatory pathway of ABA on *AchnKCS* involved in suberin biosynthesis (Fig. 7). *AchnKCS* promoter was activated by the interaction with AchnMYB70 but suppressed by the interaction with AchnbZIP29. The transcription level of *AchnMYB70* was induced by ABA, but *AchnbZIP29* expression was reduced by ABA. Therefore, ABA played a key role in the transcriptional activation of *AchnKCS* possibly by up-regulating *AchnMYB70* expression and down-regulating *AchnbZIP29* expression.

## Supplementary Information

**Additional file 1: Supplementary Table 1**. The sequences of primers used in this study.

## Data Availability

All data generated or analyzed during this study are included in this article (and its supplementary information files) or are available from the corresponding author on reasonable request.

## Abbreviations

ABA: Abscisic acid
AbA: Aureobasidin A
ABRE: ABA responsive element
AREB: ABRE binding proteins
CDS: Coding sequence
CTAB: Cetyltrimethylammonium bromide
FAE: Fatty acid elongation enzyme complex
GFP: Green fluorescent protein
KCS: β-ketoacyl-coenzyme A synthase
LUC: Firefly luciferase
NCBI: National Center of Biotechnology Information
qRT-PCR: Real-time quantitative reverse transcription PCR
REN: Renilla luciferase
VLCFAs: Very long chain fatty acids
Y1H: Yeast one-hybrid
