# Supplementary material for: Transcriptional regulation of KCS gene by bZIP29 and MYB70 transcription factors during ABA-stimulated wound suberization of kiwifruit (Actinidia deliciosa)
Source: BMC Plant Biol. 2022 Jan 8;22:23. doi: 10.1186/s12870-021-03407-6 (PMC8742354; doi:10.1186/s12870-021-03407-6)
Supplement: Supplementary file 1 — Additional file 1: Supplementary Table 1. The sequences of primers used in this study. [file 12870_2021_3407_MOESM1_ESM.docx]

**Supplementary Table 1** Primers used in this study

| Gene | Forward primer (5ˊ-3ˊ) | Reverse primer (5ˊ-3ˊ) | Accession No. |
| --- | --- | --- | --- |
| *Actin* | GTGCTCAGTGGTGGTTCAA | GACGCTGTATTTCCTCTCAG | Achn107181 |
| *AchnKCS-Pro* | AAATGTAGGGGGCAAATTGA | TGACTCGGCCCATTAACGAC | Achn030011 |
| *AchnKCS–Pro*-*Y1H* | TCCCCCGGGAAATGTAGGGGGCAAATTGA | ACGCGTCGACTGACTCGGCCCATTAACGAC |  |
| *AchnKCS-Pro*-LUC | GTCGACGGTATCGATAAGCTTAAATGTAGGGGGCAAATTGA | CGCTCTAGAACTAGTGGATCCTGACTCGGCCCATTAACGAC |  |
| *AchnbZIP*29*-Full* | ATGGGAGATACTGAGGAAGCTCGT | CTACTGGTTTGAGTCTTGCTTTGT | Achn340751 |
| *AchnMYB*70-*Full* | ATGAGCGCCGATGACGTAAAC | CTACTCCATCTCGCTAATTCCTATGC | Achn117821 |
| *AchnbZIP*29*-RT* | GCGATCCAATAGTGATATTC | GTCATCCACAACTTCTCCTT |  |
| *AchnMYB*70-*RT* | TGGTTCCAGTGAAGCATCTC | TTCCACACCTCCGTCCTTAT |  |
| *AchnbZIP*29*-GFP* | TCCCCCGGGTATGGGAGATACTGAGGAAGCTCGT | GTCGACCTGGTTTGAGTCTTGCTTTGT |  |
| *AchnMYB*70-*GFP* | CGAGCTCATGAGCGCCGATGACGTAAAC | TCCCCCGGGTCTCCATCTCGCTAATTCCTATGC |  |
| *AchnbZIP*29-*AD* | GGAATTCATGGGAGATACTGAGGAAGCTCGT | CCGCTCGAGCTACTGGTTTGAGTCTTGCTTTGT |  |
| *AchnMYB*70*-AD* | TCCCCCGGGATGAGCGCCGATGACGTAAAC | CGAGCTCCTACTCCATCTCGCTAATTCCTATGC |  |
| *AchnbZIP*29-*SK* | GCGGCCGCTCTAGAACTAGTGATGGGAGATACTGAGGAAGC | GGTCGACGGTATCGATAAGCTCTACTGGTTTGAGTCTTGCT |  |
| *AchnMYB*70*-SK* | GCGGCCGCTCTAGAACTAGTGATGAGCGCCGATGACGTA | GGTCGACGGTATCGATAAGCTCTACTCCATCTCGCTAAT |  |

Note: Accession No. is from Cornell University kiwifruit genome database.
